# Implication of Extracellular Signal-Regulated Kinase in the Expression of Natural Reward: Evidence Not Found

**DOI:** 10.3389/fnbeh.2022.856675

**Published:** 2022-03-18

**Authors:** Inês M. Amaral, Alex Hofer, Rana El Rawas

**Affiliations:** Division of Psychiatry I, Department of Psychiatry, Psychotherapy, Psychosomatics and Medical Psychology, Medical University Innsbruck, Innsbruck, Austria

**Keywords:** drugs of abuse, natural reward, social interaction, nucleus accumbens, ERK

## Abstract

Many studies have implicated extracellular signal-regulated kinase (ERK) in drug-rewarding properties. Yet, only few investigated whether ERK also mediates the naturally rewarding stimuli. In this study, we compared ERK activation in the nucleus accumbens (NAc) after cocaine reward and after positive social interaction (SI) with a partner-reward in male rats. With our protocol, ERK phosphorylation in the NAc was not increased after cocaine reward. In addition, the interaction with a social partner did not alter ERK activation in the NAc. These results suggest that ERK in the NAc may not be involved in natural reward learning. SI in an alternative context to the one associated with drugs of abuse can abolish drug preference. Given that intra-NAc core ERK inhibition impaired the expression of cocaine preference, we wanted to investigate whether the protective effects of SI when an individual is allowed to interact with a social partner in an alternative context to the one associated with drugs during the learning phase are enhanced by ERK inhibition. For that, U0126 was bilaterally infused into the NAc core of rats conditioned with cocaine in one context and with SI in the opposite context before assessing the expression of reward-related learning. Intra-NAc core ERK inhibition was ineffective to impair the expression of drug reward as previously demonstrated, when a social partner was available in an alternative context. Thus, the effects of the pharmacological manipulations based on decreasing ERK activity are not cumulative to other treatments for drug addiction based on SI.

## Introduction

Impaired social interaction (SI) is a common feature of drug-dependent individuals. In substance use disorders, impaired SI incites addicts to increase their drug consumption and restricts recovering addicts to adhere to treatment based on dyadic SI with healthcare providers (Zernig and Pinheiro, 2015). Thus, shifting the preference of drug-dependent individuals toward natural reward such as positive SI represents a challenge in drug therapy.

When SI is made available as an alternative choice to drugs, operant social reward has been shown to prevent drug self-administration in rats (Venniro et al., 2018, 2021). Moreover, pairing one compartment with cocaine and the opposite to a social stimulus during the conditioning sessions of the conditioned place preference (CPP) paradigm yielded to a similar preference (Fritz et al., 2011). These findings suggest that dyadic SI and cocaine have the same conditioned reward value and that the presence of a social partner in an opposite context to drugs abolishes drugs’ preference. Furthermore, after establishment of cocaine conditioning, mice that had the option to choose between a cocaine-paired compartment and a compartment where an unfamiliar juvenile mouse was placed during the CPP test in the non-cocaine paired compartment spent more time in the social-paired compartment (Sampedro-Piquero et al., 2019).

Even though both natural and drug reward are modulated by the nucleus accumbens (NAc) (Kelley, 2004), different intracellular cascades might be involved in the acquisition and expression of learning produced by either type of stimuli (Gerdjikov et al., 2007). It has been reported that protein kinase A (PKA) in the NAc is involved in the expression of drug but not natural reward-associated behaviors (Self et al., 1998; Baldwin et al., 2002; Misra and Pandey, 2006; Gerdjikov et al., 2007; Amaral et al., 2021). In addition, it was found that a bilateral infusion of Rp-cAMPS, a specific PKA inhibitor, directly into the NAc, shifted rats’ preference toward cocaine when a social partner was made available in the alternative compartment of the CPP (Amaral et al., 2021).

Many studies have focused on the involvement of the extracellular signal-regulated kinase (ERK), a member of the mitogen-activated protein kinase family (MAPK), in cocaine-rewarding properties (Valjent et al., 2000, 2006; Miller and Marshall, 2005); however, the effects of natural reward on the subsequently activated ERK expression are underinvestigated. In this study, we compared the levels of phosphorylated ERK after drug reward, particularly cocaine, and after SI natural reward. We hypothesized that after cocaine CPP, levels of activated ERK would be enhanced as compared to those after SI CPP. In another set of experiments, and based on findings reporting that inhibition of ERK before the CPP test impaired the expression of cocaine CPP (Miller and Marshall, 2005; Valjent et al., 2006), we wanted to explore whether the protective effects of SI when available as an alternative to drugs are enhanced by an additional ERK inhibition. For that, rats received a bilateral intra-NAc infusion of U0126, a MAPK/ERK kinase (MEK) inhibitor before the CPP test of a concurrent CPP paradigm, in which a social partner was made available in the alternative compartment to the one associated with cocaine. We proposed that rats would shift their preference toward the social partner-associated compartment in comparison with intra-NAc vehicle-infused rats that would express similar preference to the cocaine and the social partner-paired compartments.

## Materials and Methods

### Animals

Male Sprague-Dawley rats aged 6–7 weeks (150–250 g) were obtained from Janvier Labs, France. The animals were housed at a constant room temperature and had *ad libitum* access to water and pellet chow. Animals were isolated upon the arrival to the animal facility and remained singly housed during the entire experiment. Indeed, it was shown that social play behavior peaks in isolated adolescent male rats (Douglas et al., 2004) as a result of a high motivation for SI due to isolation (Trezza et al., 2009). These experiments were performed during the light phase of a continuous 12-hlight–dark cycle (from 8:00 to 20:00). All animals were 8 weeks old when starting the experimental behavioral tests. All animal procedures were performed in accordance with the Austrian National Animal Experiment Ethics Committee [permit numbers BMWF-66.011/0131-WF/V/3b/2016 and BMWF-66.011/0040-WF/V/3b/2019].

### Place Conditioning Procedure

Conditioning was performed in a three-compartment apparatus (64 cm wide × 32 cm deep × 31 cm high). The middle (neutral) compartment had white walls and a white floor. Two doors connected the middle compartment to the two conditioning compartments, with walls displaying either vertical or horizontal black-and-white stripes of the same overall brightness, and stainless-steel floors containing either holes or slits.

The trajectory of the animal was recorded with a video camera placed above the apparatus and analyzed with the ANY-maze Video Tracking Software. The time the animals spent in the compartments and the distance in the cocaine-associated chamber were evaluated. After each session, the CPP apparatus was cleaned with 70% camphorated ethanol solution.

#### Acquisition of Place Preference

**FIGURE 1:**
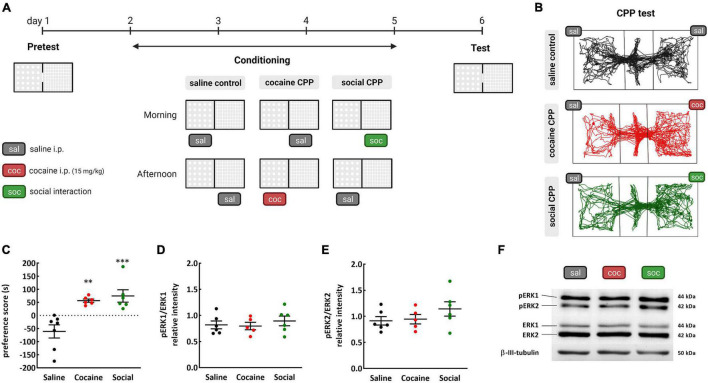
Conditioned place preference (CPP) to cocaine or SI; **(A)** timeline; **(B)** representative tracking of rats receiving saline injections in both compartments of the CPP (saline controls), and rats conditioned to cocaine (coc–cocaine CPP) or SI (soc–social CPP) in each compartment during the CPP test; **(C)** preference score is calculated based on the time the rats spend in the stimulus-associated compartment during the test minus the pretest; **(D)** pERK 1 and **(E)** pERK 2 relative intensity of expression in the NAc of rats after the test of CPP to cocaine or SI; **(F)** representative western blot images of phosphorylated ERK1 and 2, ERK1 and 2 and β-III tubulin levels (used as protein loading control) in the NAc of rats from the saline, cocaine, and social CPP expressing rats. ^**^p < 0.01and ^***^p < 0.001 Tukey’s multiple comparisons test, different from the saline control group, *n* = 5–7/group.

The acquisition protocol comprised a pre-test session on day 1, in which the animals were allowed to freely explore the three compartments of the CPP. After day 1, rats underwent four consecutive conditioning days from days 2 to 5 (two sessions/day) separated by at least 4 h, with a total of four conditionings/stimulus (Figure 1A). For cocaine and SI CPP groups, the less preferred compartment in the pre-test was paired with cocaine or with a social partner in the conditioning sessions. The opposite compartment was paired with saline. Cocaine or saline was injected intraperitoneally (i.p.) immediately before placing the rat in the compartment.

Cocaine (hydrochloride salt) was dissolved to a concentration of 15 mg/kg of pure cocaine base in a volume of 1 mL/kg of saline solution. A control group, that is, the saline CPP group, received i.p. saline in both compartments of the CPP.

In the social conditioning sessions, each rat received an i.p. injection of saline and was placed in the associated compartment together with a conspecific partner of the same weight and sex, which was assigned in the first conditioning and remained the same for the whole duration of the experiment. Both animals remained singly housed. On day 6, animals were tested for CPP by being placed in the middle compartment of the apparatus and allowed to move freely between all the compartments of the CPP.

All sessions were of equal duration, that is, 900 s. The preference score was calculated as the time in the SI or cocaine-associated compartment in the test minus the time spent in the same compartment in the pre-test session. For the saline control group, the preference score was calculated as the time in the less-preferred compartment during the test minus the pre-test.

#### Concurrent Conditioned Place Preference Paradigm

**FIGURE 2:**
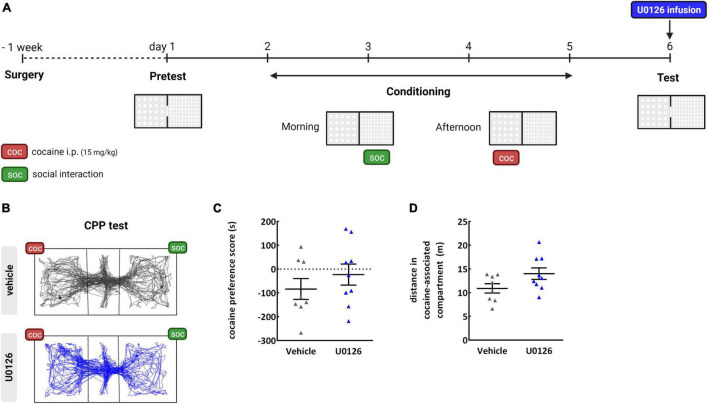
Inhibition of ERK in the NAc by a bilateral infusion of U0126; **(A)** timeline; **(B)** representative tracking of rats receiving cocaine injections in one compartment and the opportunity of SI with a partner in the opposite compartment, after vehicle or bilateral U0126 intra-NAc infusions. **(C)** Cocaine preference score in a concurrent paradigm in which rats were conditioned to cocaine at the dose of 15 mg/kg and to SI in the opposite compartment. Intra-NAc U0126 bilateral infusions before the CPP test did not affect cocaine preference; **(D)** distance in cocaine-associated compartment during the test after U0126 infusions was trending to increase (*p* = 0.07) without reaching significance as compared to vehicle-infused rats, *n* = 8–9/group.

Animals were concurrently trained for CPP by pairing cocaine with one compartment and a social partner with the other compartment. The protocol comprised a pre-test on day 1, followed by four conditioning days (two sessions/day) for each stimulus from days 2 to 5 (social session performed in the morning and cocaine session in the afternoon), and a CPP test on day 6 (Figure 2A).

All sessions were of equal duration (900 s). Results were expressed as cocaine preference score.

### Surgeries and Intra-Nucleus Accumbens Infusions

Rats were 7 weeks old when they underwent surgery. Briefly, guide cannulae (Plastics One, 23G, Bilaney) were implanted bilaterally in the NAc region (anteroposterior: ± 1.6 mm, mediolateral: ± 2.3 mm, dorsoventral: −7.2 mm relative to bregma) (Supplementary Figure 1), for infusion of the MEK inhibitor or vehicle following the procedure described in the study by Miller and Marshall (2005). Following surgery, the animals received post-operative analgesia [a daily intraperitoneal injection of meloxicam (Metacam, 5 mg/ml) diluted in saline (dose 1 mg/kg) for up to 3 days post-surgery] and were allowed to recover for at least 5 days before the behavioral experiments.

U0126 (Tocris) was dissolved in 5% dimethylsulfoxide (DMSO) in 6% Tween 80 in saline to a concentration of 2 μg/μl (Miller and Marshall, 2005). On the test day, animals received an infusion of 0.5 μl/side of vehicle or U0126 (1 μg/0.5 μl/side), over a period of 2 min. Then, 30 min after the infusion, rats were tested for CPP. In this experiment, half of the rats that belong to the vehicle group received an infusion of 0.9% sterile saline solution and the other half received an infusion of 5% DMSO in 6% Tween 80 in saline. Since we did not observe any behavioral difference between these rats, they were merged into one vehicle group.

### Western Blot

Rats were sacrificed 20 min after the end of the CPP test by an overdose of CO2 inhalation. The brains were removed and immediately frozen in isopentane in dry ice. Tissue punches from the NAc were collected from thaw-mounted 150-μm coronal sections obtained in a cryostat using a sample corer and frozen at −80°C until used.

In experiments where cannulae were implanted in the NAc for the infusion of the MEK inhibitor, cannulae placement was confirmed during this step. In Supplementary Figure 1, we show representative coronal sections of the NAc, with infusion sites of vehicle and U0126. All infusion sites were located in the core subdivision of the NAc (Miller and Marshall, 2005).

Total protein was extracted from the collected NAc tissue. Then, protein samples (20 μg) were prepared for SDS-PAGE by adding Roti-Load buffer (Lactan, Graz, Austria), loaded onto 10% acrylamide gels, and then transferred to PVDF membranes. After a blocking step, the membranes were incubated with primary antibodies, ERK1/ERK2 (#4695, Cell Signaling, 1:1,000), pERK1/pERK2 (#4377, Cell Signaling, 1:1,000), and β-III Tubulin (#NB100-1612, Novus Biologicals, 1:50,000), used as a loading control. Blots were then incubated with the secondary antibodies. Finally, blots were developed in a Chemidoc Imaging System (Bio-Rad) after incubation with an enhanced chemiluminescence substrate (Bio-Rad). Results were expressed as relative intensity of the ratio between phosphorylated and total protein, which were previously normalized to tubulin. Image Lab Software (Bio-Rad) was used to quantify the band intensity.

### Statistical Analyses

Statistical analyses were performed using GraphPad PRISM (GraphPad Software; CA, United States). All data were expressed as mean ± standard error of the mean (SEM), and *p*-values < 0.05 were considered statistically significant. Before the use of parametric analysis, all the data were tested for normality and were found to pass the normality tests. The significance between two experimental groups was tested using a two-tailed unpaired Student’s *t*-test. To test the statistical difference between three groups, one-way analysis of variance (ANOVA) was used, followed by Tukey’s multiple comparisons *post-hoc* test. Cohen’s d was calculated to evaluate effect sizes. A Cohen’s *d*-value higher than 0.8 is considered a “large” effect size.

## Results

### Conditioned Place Preference to Social Interaction Does Not Increase Extracellular Signal-Regulated Kinase Phosphorylation in the Nucleus Accumbens

To explore the levels of ERK phosphorylation in the NAc following natural vs. drug reward, first rats were trained to express cocaine or social CPP. Control rats received saline i.p. injections in both compartments of the CPP. After conditioning sessions, rats expressed equally cocaine or social CPP [one-way ANOVA, effect: treatment, *F*_(2_,_16)_ = 13.03, *p* = 0.004; Tukey’s multiple comparisons test: saline vs. cocaine, *p* < 0.01, Cohen’s *d* = 1.313256; saline vs. social, *p* < 0.001, Cohen’s *d* = 0.099056; cocaine vs. social, *p* > 0.05 non-significant (n.s)] (Figures 1B,C).

Then, 20 min after the CPP test, the expression of activated ERK was evaluated in the NAc of rats expressing cocaine CPP, social CPP, and saline control. Levels of phosphorylated ERK 1 (pERK1) [one-way ANOVA, effect: treatment, *F*_(2_,_14)_ = 0.3938, *p* = 0.6817] and phosphorylated ERK 2 (pERK2) [one-way ANOVA, effect: treatment, *F*_(2_,_14)_ = 1.354, *p* = 0.2901] in the NAc were unaltered after CPP to cocaine or to SI (Figures 1D–F). Raw data for pERK1 and pERK2 relative intensities are detailed in Supplementary Table I. These data suggest that neither drug reward nor natural reward was able to increase ERK activation in the NAc.

### Social Interaction Protective Effects Are Not Enhanced by an Intra-Nucleus Accumbens Core Extracellular Signal-Regulated Kinase Inhibition Before the Conditioned Place Preference Test

To investigate the effects of ERK inhibition in the NAc on cocaine preference when SI is available in an alternative context, U0126 was infused bilaterally in the NAc before the CPP test (24 h after the last conditioning session).

The vehicle-infused group showed no preference for the cocaine-associated compartment when SI was offered in the compartment opposite to the one associated with cocaine (Figures 2B,C). The bilateral infusion of U0126 in the NAc core did not affect the expression of cocaine preference in the concurrent paradigm [unpaired student’s *t*-test, two-sided, effect: treatment, U0126 vs. vehicle, *t*_(15)_ = 0.9723, *p* = 0.3463] (Figures 2B,C).

When comparing the distance in the cocaine-associated compartment during the test, a trend for an increase in locomotion was observed in the intra-NAc core U0126-infused group as compared to the vehicle-infused group [unpaired student’s *t*-test, two-sided, effect: treatment U0126 vs. vehicle, *t*_(15)_ = 1.948, *p* = 0.0704] (Figure 2D). Furthermore, the number of entries in the cocaine and the SI-associated compartments in the CPP test were not different between the U0126 and the vehicle-infused groups (Supplementary Table II).

## Discussion

We found that natural reward does not affect ERK phosphorylation in the NAc. Indeed, our results show that ERK expression was not altered in the NAc after CPP to natural reward such as SI nor after CPP to cocaine at a dose of 15 mg/kg. Moreover, intra-NAc infusion of U0126 did not alter the expression of CPP in a concurrent paradigm for SI vs. cocaine, which suggests that the protective effects of SI were not enhanced by ERK inhibition.

Many studies have reported an increase in ERK activation in the NAc after cocaine CPP. For example, one day after the CPP test to cocaine, a reconditioning with cocaine in the cocaine-paired compartment has been shown to increase phospho-ERK2 levels in the NAc (Valjent et al., 2006; Marion-Poll et al., 2019). These findings suggest that a reexposure to both cocaine and a drug-paired context is required to activate ERK in the NAc. Yet, reexposure only to the cocaine-paired compartment yielded to activated ERK in the NAc when the reexposure occurred one day after the CPP test (Nygard et al., 2015) but not two days after the CPP test (Tropea et al., 2008). When evaluated directly after the CPP test without reexposure to cocaine or to the cocaine-associated context, ERK was activated in the NAc of animals conditioned with the drug at the dose of 20 mg/kg (Valjent et al., 2006; Nygard et al., 2013, 2017). On the other hand, phospho-ERK was reported to increase only in the core but not the shell subdivision of the NAc after the CPP test to cocaine at a dose of 10 mg/kg (Miller and Marshall, 2005). In our study, rats were trained with cocaine at a dose of 15 mg/kg. Potentially, this dose may not be sufficient to induce ERK phosphorylation in the NAc directly after the CPP test without reexposure to cocaine or to the cocaine-associated context. In addition, our data reflect ERK phosphorylation levels examined in the rats’ both core and shell subdivisions of the NAc, which could have masked any slight subregion-specific alteration.

Conditioned place preference to natural reward such as SI did not affect ERK phosphorylation in the NAc, thereby suggesting that natural reward preference does not involve ERK activation. In line with these findings, ERK2 activation in the NAc has previously been found to be increased in rats trained with a tone as a conditioned stimulus predicting food delivery in comparison with rats trained to the context only (Shiflett et al., 2008). Moreover, sexual behavior alone did not induce ERK phosphorylation in the NAc core or shell in a study by Frohmader et al. (2010).

Consistent with the ERK intracellular signaling pathway role in learning and memory, pretreatment with SL 327, a pharmacological inhibitor of MEK, during the conditioning phase, completely abolished cocaine CPP in a study by Valjent et al. (2000). In addition, the deletion of the ERK1 isoform, which leads to increased ERK2 stimulus-dependent signaling, has been shown to facilitate the acquisition of cocaine CPP (Ferguson et al., 2006). These findings emphasized the implication of ERK signaling in the acquisition of cocaine preference. Interestingly, pretreatment with SL 327 before cocaine reexposure in the drug-paired environment has been demonstrated to be sufficient to abolish the previously acquired cocaine CPP (Valjent et al., 2006). We investigated the effects of U0126 infusion on the expression of a concurrent CPP paradigm in which one compartment was paired with cocaine and the opposite compartment was paired with a social partner. It has previously been shown that a pretreatment with a peripherally administered SL 327 (Valjent et al., 2006) or PD325901 (Papale et al., 2016) decreases the expression of cocaine CPP. Furthermore, bilateral intra-NAc infusion of U0126 before the CPP test impaired the expression of cocaine CPP in a study by Miller and Marshall (2005). Therefore, as we followed the procedure described by Miller and Marshall (2005), we expected that rats infused by U0126 in the NAc would shift their preference toward SI if the protective effects of SI are cumulative to the effects associated with ERK inhibition before the CPP test. However, we found that both vehicle- and U0126-infused groups expressed similar preference to cocaine and SI. The lack of effects in our study might be due to our design as we used a concurrent protocol, whereas in the study of Miller and Marshall (2005), a normal CPP procedure was performed. It appears that NAc core ERK mediates the expression of cocaine reward only when saline is present as an alternative to drugs. Yet, when an alternative reward of the same strength is made available during the conditioning sessions, other kinases, rather than ERK, seem to get involved in cocaine reward expression (Amaral et al., 2021). Furthermore, when SI is available in an alternative context, ERK inhibition effects are not summed up with SI protective effects but rather, SI masks the effects of ERK inhibition on the expression of drug reward learning. Another discrepancy between our protocol and the one in the study of Miller and Marshall (2005) lays in the length of the conditioning protocols. In the latter study, the conditioning consisted of 30-min sessions over a period of 6 days in an alternate day design (three cocaine pairings) compared to our conditioning sessions (15 min over a period of 4 days with morning and afternoon sessions and a total of four cocaine pairings). Although unlikely, it is not impossible that ERK inhibition efficiency might be determined by how the drug conditioning was performed.

One limitation of this study is that ERK inhibition was performed only before the CPP test, thereby focusing on the expression of reward-related learning. Interestingly, infusion of U0126 into the ventral tegmental area (VTA) during the acquisition phase of the CPP paradigm, but not before the CPP test, has been shown to attenuate cocaine-induced CPP, thereby suggesting that ERK activity is required for the acquisition, but not the expression of CPP to cocaine (Pan et al., 2011). Indeed, ERK activity in the VTA, which is most likely required for the establishment of an association between environmental cues and rewarding effects of cocaine, is not necessary for maintenance of the expression of cue-associated memories once such an association is established (Pan et al., 2011). Possibly, other brain regions such as the NAc may be recruited to maintain the expression of CPP to cocaine (Miller and Marshall, 2005; Pan et al., 2011). The previous research has shown that both cocaine and SI CPP induce an activation of the NAc (Rawas et al., 2012). Hence, in a concurrent paradigm, both stimuli would engage the NAc to maintain an equal expression of preference for SI and cocaine reward, thereby supporting a lack of ERK inhibition in reward expression when both stimuli are offered.

Pharmacological manipulations that decrease the extent to which cocaine and cocaine cues induce ERK activity have been suggested as potential treatments for cocaine addiction (Lu et al., 2006; Papale et al., 2016). Yet, it seems that these manipulations should not be combined with other treatments based on dyadic SI between treatment-seeking individuals and various healthcare providers such as psychiatrists, psychotherapists, or social workers if applied after that drug reward learning already occurred.

## Data Availability Statement

The original contributions presented in the study are included in the article/Supplementary Material, further inquiries can be directed to the corresponding author.

## Ethics Statement

The animal study was reviewed and approved by the Austrian National Animal Experiment Ethics Committee (Permit numbers BMWF-66.011/0131-WF/V/3b/2016 and BMWF-66.011/0040-WF/V/3b/2019).

## Author Contributions

RE designed the research. IA performed the research. IA and RE analyzed the data. RE, IA, and AH wrote and edited the manuscript. All authors contributed to the article and approved the submitted version.

## Conflict of Interest

The authors declare that the research was conducted in the absence of any commercial or financial relationships that could be construed as a potential conflict of interest.

## Publisher’s Note

All claims expressed in this article are solely those of the authors and do not necessarily represent those of their affiliated organizations, or those of the publisher, the editors and the reviewers. Any product that may be evaluated in this article, or claim that may be made by its manufacturer, is not guaranteed or endorsed by the publisher.
