# Supplementary material for: Implication of Extracellular Signal-Regulated Kinase in the Expression of Natural Reward: Evidence Not Found
Source: Front Behav Neurosci. 2022 Mar 18;16:856675. doi: 10.3389/fnbeh.2022.856675 (PMC8973696; doi:10.3389/fnbeh.2022.856675)
Supplement: Supplementary file 1 [file Data_Sheet_1.docx]

Supplementary Material

**Suppl. Table I:** quantification of pERK1, ERK1 and pERK2 and ERK2 bands relative intensity.

| **Animal** | **treatment** | **pERK1*** | **ERK1*** | **pERK1/ERK1** | **pERK2*** | **ERK2*** | **pERK2/ERK2** |
| --- | --- | --- | --- | --- | --- | --- | --- |
| #1 | Saline | 0.569 | 0.855 | 0.666 | 0.577 | 0.728 | 0.792 |
| #2 | Saline | 0.535 | 0.738 | 0.725 | 0.489 | 0.586 | 0.835 |
| #3 | Saline | 1.014 | 1.082 | 0.937 | 0.981 | 1.156 | 0.849 |
| #4 | Saline | 0.895 | 0.793 | 1.129 | 0.776 | 0.630 | 1.231 |
| #5 | Saline | 0.636 | 0.970 | 0.656 | 0.575 | 0.824 | 0.697 |
| #6 | Saline | 0.749 | 0.938 | 0.798 | 0.757 | 0.699 | 1.082 |
| **MEAN** | **Saline** | **0.733** | **0.896** | **0.819** | **0.692** | **0.771** | **0.914** |
| #7 | Cocaine | 0.725 | 0.733 | 0.989 | 0.728 | 0.686 | 1.060 |
| #8 | Cocaine | 0.564 | 0.735 | 0.767 | 0.495 | 0.531 | 0.933 |
| #9 | Cocaine | 0.690 | 0.963 | 0.717 | 0.631 | 0.891 | 0.708 |
| #10 | Cocaine | 0.737 | 0.799 | 0.922 | 0.636 | 0.522 | 1.219 |
| #11 | Cocaine | 0.656 | 1.104 | 0.594 | 0.658 | 0.813 | 0.809 |
| **MEAN** | **Cocaine** | **0.674** | **0.867** | **0.798** | **0.630** | **0.689** | **0.946** |
| #12 | Social | 0.948 | 0.780 | 1.215 | 0.905 | 0.540 | 1.675 |
| #13 | Social | 0.571 | 0.705 | 0.811 | 0.542 | 0.523 | 1.038 |
| #14 | Social | 0.972 | 0.966 | 1.007 | 0.934 | 0.770 | 1.212 |
| #15 | Social | 1.028 | 1.013 | 1.014 | 1.055 | 0.825 | 1.278 |
| #16 | Social | 0.681 | 0.930 | 0.732 | 0.681 | 0.701 | 0.971 |
| #17 | Social | 0.635 | 1.079 | 0.589 | 0.615 | 0.908 | 0.677 |
| **MEAN** | **Social** | **0.806** | **0.912** | **0.895** | **0.789** | **0.711** | **1.142** |

*Normalized to ß-III-tubulin.

**
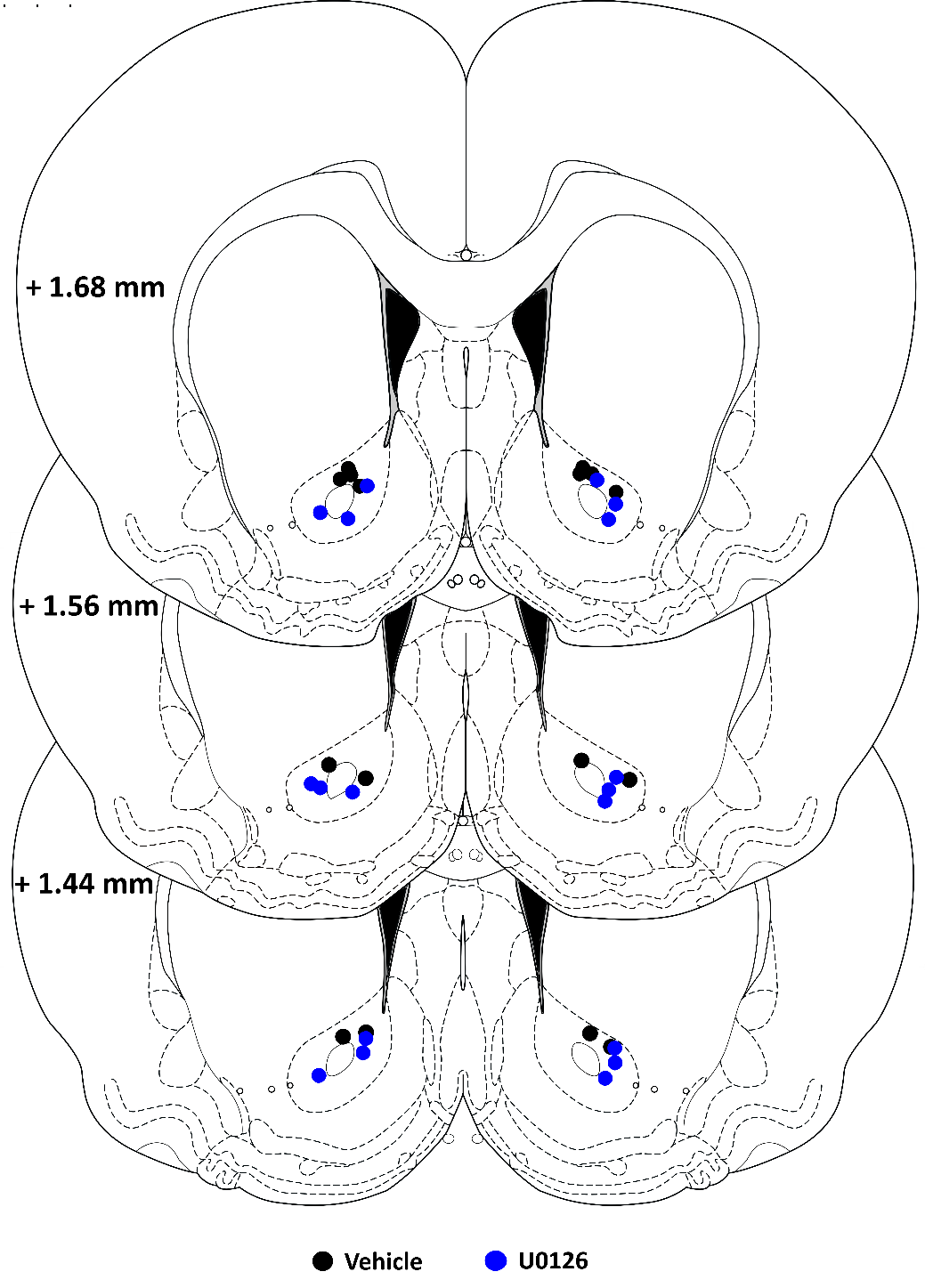
Suppl. Figure 1:** Representative image for the infusion sites in the nucleus accumbens core for the U0126 and the vehicle infused rats.

**Suppl. Table II:** Number of entries in the cocaine and social interaction-associated compartments in the CPP test.

| Animal | Treatment | Cocaine | Social interaction |
| --- | --- | --- | --- |
| #18 | Vehicle | 25 | 19 |
| #19 | Vehicle | 26 | 23 |
| #20 | Vehicle | 31 | 32 |
| #21 | Vehicle | 15 | 19 |
| #22 | Vehicle | 40 | 55 |
| #23 | Vehicle | 32 | 26 |
| #24 | Vehicle | 28 | 29 |
| #25 | Vehicle | 25 | 25 |
| #26 | U0126 | 32 | 40 |
| #27 | U0126 | 40 | 46 |
| #28 | U0126 | 30 | 41 |
| #29 | U0126 | 35 | 39 |
| #30 | U0126 | 24 | 33 |
| #31 | U0126 | 24 | 34 |
| #32 | U0126 | 21 | 20 |
| #33 | U0126 | 34 | 28 |
| #34 | U0126 | 28 | 25 |
